# Acute Rheumatic Fever in Caucasians: A Case Report and Systematic Review

**DOI:** 10.3390/life15071131

**Published:** 2025-07-18

**Authors:** Fuad Hasan, Mrinalini Dey, Arvind Nune

**Affiliations:** 1 Mersey & West Lancashire Teaching Hospital NHS Trust, Southport PR8 6PN, UK; fuad.hasan@merseywestlancs.nhs.uk; 2 Centre for Rheumatic Diseases, Weston Education Centre, Cutcombe Road, King's College London, London SE5 9RJ, UK;mrinalini.dey@kcl.ac.uk

**Keywords:** rheumatic fever, Caucasians, systematic review, adults, developed world, chorea, erythema mariginatum, rheumatic heart disease, group A streptococcus

## Abstract

Acute Rheumatic Fever (ARF) is more common in children in the developing world. The current incidence in the United Kingdom is reported to be less than 1 in 100,000 children. It is, however, rare in the developed world, particularly in the adult Caucasian population. We present a case of ARF in a 39-year-old Caucasian female who needed multiple hospital admissions before the ARF diagnosis was made. A comprehensive, up-to-date literature review of ARF in Caucasians is lacking. Therefore, a systematic literature review (SLR) of Medline, PubMed, and Google Scholar was conducted to investigate the characteristics, management, and prognostic outcomes of new cases of ARF among Caucasians. A total of 10 cases were reported from six countries between 1990 and 2022. The mean age of patients was 33.2 (range 18–41), and most were females (6, 60%). The most common presenting symptoms were fever, arthralgia, and malaise. All patients met the modified Jones criteria for ARF diagnosis. All patients received antibiotics, with only one patient requiring corticosteroids. Two patients developed rheumatic heart disease (RHD), and none died as a result of ARF. This case-based literature review underscores the critical importance of a high index of clinical suspicion in promptly diagnosing ARF to mitigate long-term sequelae of RHD.

## 1. Introduction

Acute Rheumatic Fever (ARF) is a severe inflammatory condition that can develop as a complication of untreated or inadequately treated streptococcus pyogenes throat infection or scarlet fever [1]. Although it primarily affects children and adolescents, ARF can occur at any age, leading to long-term damage to the heart, joints, and nervous system, if not promptly diagnosed and treated [2]. The underlying cause of ARF has been demonstrated to be an immunological reaction to group A streptococcus bacterial infection; however, the exact mechanisms by which the immune system attacks healthy tissues following a strep infection are not fully understood [3]. It is believed that the bacteria trigger an autoimmune reaction, causing the immune system to mistakenly target specific tissues in the body, particularly the heart and joints [4]. The diagnosis of ARF is based on a combination of clinical criteria, blood tests, and imaging tests such as echocardiography [5]. Despite the availability of data on rheumatic fever (ARF) cases within the developed world, the existing literature predominantly focuses on non-Caucasian populations, revealing a significant gap in the investigation of ARF cases among Caucasians. Consequently, there is a pressing need to examine the incidence, management strategies, and clinical outcomes related to ARF within this demographic. This systematic literature review (SLR) endeavors to critically analyze and synthesize the clinical characteristics and demographic factors pertaining to newly diagnosed ARF cases in the adult Caucasian population.

## 2. Case Presentation

This is a case of a 39-year-old female with a past medical history of Bell’s Palsy, who presented to the local accident and emergency (A&E) department at the end of 2022 with a 3-week history of fever, sore throat, arthralgia, myalgia, chest pain, and dyspnea. Her young school-aged daughter had a two-week history of fever, sore throat, generalized body aches, and fatigue, which were resolved spontaneously.

During her initial presentation, her blood tests showed raised C-reactive protein (CRP) at 171 (0–4 mg/L), lymphopenia, and abnormal liver function tests.

The test results are summarized in Table 1.

**Figure 1:**
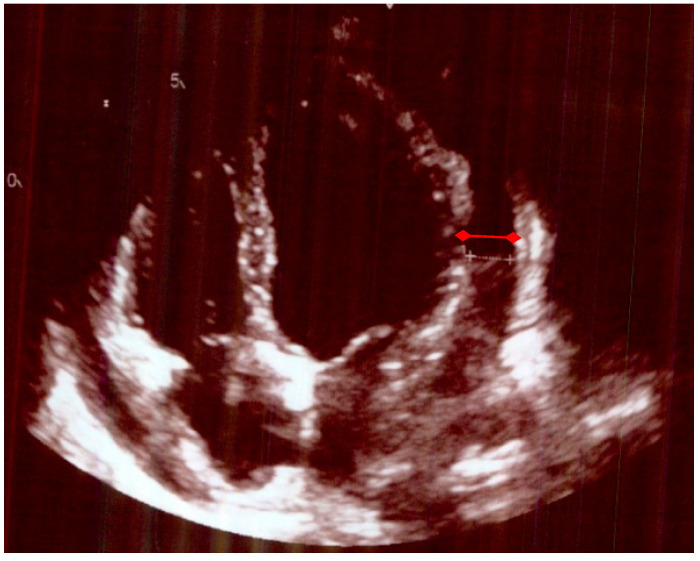
Echocardiogram, with evidence of pericardial effusion (red arrows).

As the patient’s symptoms were not severe enough to warrant hospitalization, the patient was discharged home from A&E with a suspected viral infection. A week later, the patient returned to the A&E with worsening chest pain, dyspnea, and polyarthritis in the hands and feet. Her ECG was normal. Her blood tests showed worsening of CRP at 191, hemoglobin 97 (115–165 g/L), and gamma-glutamyl transferase (GGT) at 115 (0–39 u/L). Her echocardiogram showed moderate pericardial effusion (Figure 1) and an ejection fraction (EF) of 55% without valvular involvement. The ultrasound scan of her liver and gall bladder was normal. She had negative connective tissue disease, anti-neutrophilic cytoplasmic antibody (ANCA), cyclic citrullinated peptide (CCP) antibodies, and rheumatoid factor. Her complement (C3) was slightly raised, but C4 was normal. The patient was prescribed Ibuprofen for her pericarditis and arthralgia and was discharged.

**Figure 2:**
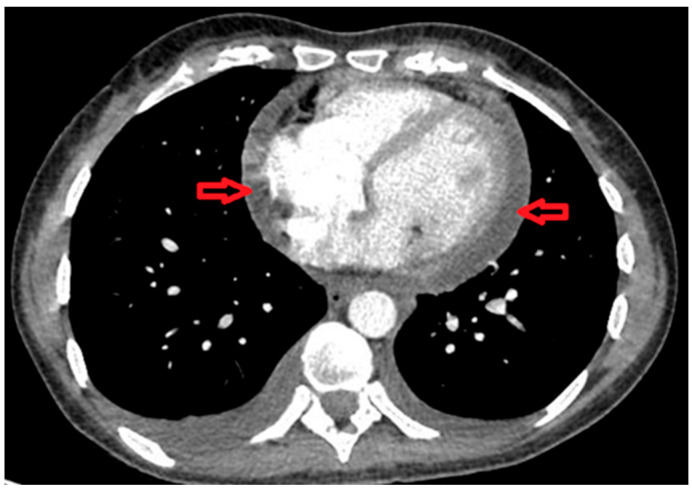
CT scan shows evidence of pericardial effusion (red arrows).

She presented to the A&E for the third time after six days with nausea, heartburn, and vomiting, which were thought to be caused by Ibuprofen. Although Ibuprofen improved her joint symptoms, it was stopped owing to the side effects, and she was started on Omeprazole. Chest computed tomography (CT) revealed moderate pericardial effusion (Figure 2) and small bilateral pleural effusions. Since her clinical features improved, she was discharged.

She returned to A&E for the fourth time 3 days later with palpitations. Her CRP and hemoglobin had improved at this visit, along with other blood parameters. Her repeat echocardiogram showed improved pericardial effusion but no changes in EF. She also had normal HIV, hepatitis C antibody, hepatitis B surface antigen, Lyme serology, Epstein–Barr virus (EBV) immunoglobulin-M, and parvovirus B19 IgM. An anti-streptolysin O titer (ASOT) was elevated at 400 (0–200); however, her throat swab was normal.

The possibility of ARF was considered by the rheumatology team when consulted, given that the patient had two major criteria (carditis and polyarthritis) and two minor criteria (elevated CRP and fever) according to the modified Jones diagnostic criteria for acute ARF (Table 2). After discussion with the infectious disease team, the patient commenced on oral Penicillin-V 500 mg 3 times a day for 10 days, followed by intramuscular Benzylpenicillin 1.2 million units 4 weekly for 10 years.

The patients’ symptoms improved gradually, and she was asymptomatic in a few weeks’ time. Her repeated echocardiogram (Table 3) with the cardiology team after a few months showed complete resolution of pericardial effusion, and EF had improved to >60%. She was completely asymptomatic on consultation.

## 3. Methods

We conducted an SLR to summarize the existing cases within the literature detailing the diagnosis and management of ARF in the Caucasian population. This SLR was conducted in accordance with the Cochrane Handbook and Preferred Reporting Items for Systematic Reviews and Meta-Analysis guidelines [6]. The search strategy is available in the Supplementary Material. The bibliographic databases Medline, PubMed, and Google Scholar were searched on 13th of May 2025, Figure 3.

No time restriction was applied to the search. Only English-language articles were included. We included adult Caucasian patients with a clinician-confirmed diagnosis of ARF. We opted to focus on the Caucasian population due to the relative paucity of data on this patient group, despite the increasing prevalence of ARF in this patient group. Only single case reports were included, with all other article types excluded. Titles and abstracts were then screened by one reviewer, with full articles that met the inclusion criteria examined in detail. Screen and data extraction were validated by a second reviewer.

Data extracted from included articles comprised (but were not limited to): demographics, presenting symptoms, organ involvement, investigations, and clinical outcome. Data were summarized using descriptive statistics.

## 4. Results

From the 106 articles retrieved (following de-duplication), 10 cases were ultimately included. The mean age was 33.2 years (SD 8.7, range 18–41), with 60% of patients being female. Cases were published between 1990 and 2022, with the following countries of origin: USA (n = 4), UK (n = 2), Australia (n = 1), Cuba (n = 1), Cyprus (n = 1), and Ireland (n = 1) (Table 4).

All patients met the modified Jones’ criteria for the diagnosis of ARF. Where reported, all patients presented with a raised C-reactive protein (range 37–503 mg/L, normal < 7), raised erythrocyte sedimentation rate (58–115 mm/h, normal ≤ 20) and positive ASOT. The most common manifestations on presentation were fever (n = 8), arthralgia (n = 6), and malaise (n = 3). One patient presented with monoarthritis, another developed an advanced atrioventricular block, and another exhibited erythema mariginatum and chorea. Two patients developed rheumatic heard disease (RHD), one of whom required admission to the intensive care unit for inotrope support [7,8].

All included patients survived following treatment with antibiotics, most frequently penicillin (n = 6). Three were given aspirin in addition, and one received prednisolone [7,9,10,11].

**Table 4:** Summary of case reports of Caucasians diagnosed with acute rheumatic fever.

SN	Article	Age	Sex	Country	Presenting Complains	Met modified Jones Criteria	Evidence of Rheumatic Heart Disease	CRP (mg/L)	ESR (mm/h)	Raised ASOT	Treatment Received	ITU Admission	Outcome
1	Farrell 1990 [9]	40	F	UK	Rash, fever, arthralgia	Yes	None	92	70	Yes	Penicillin, Aspirin	No	Survived
2	Sahi 1993 [12]	38	F	Cyprus	Malaise, flu-like symptoms	Yes	None	N/A	100	Yes	Penicillin	No	Survived
3	Barold 1996 [7]	39	M	USA	Sore throat, fever, myalgia	Yes	Yes	41	111	Yes	Penicillin, Aspirin	No	Survived
4	Grover 2009 [11]	25	M	USA	Polyarthritis, fever	Yes	None	N/A	115	Yes	Penicillin, Prednisolone	No	Survived
5	Ilgenfritz 2013 [10]	27	M	USA	Knee pain and swelling, fever	Yes	None	N/A	58	Yes	Penicillin, Aspirin	No	Survived
6	Khan 2018 [13]	41	F	Australia	Fever, polyarthritis	Yes	None	116	103	Yes	Cephalexin	No	Survived
7	Wilson 2021 [14]	24	M	UK	Shoulder pain, shortness of breath	Yes	None	200	N/A	Yes	Penicillin	No	Survived
8	Batta 2022 [15]	41	F	USA	Erythematous papules, Fever	Yes	None	37	N/A	Yes	Ceftriaxone	No	Survived
9	Our case 2022	39	F	UK	Fever, sore, throat, myalgia	Yes	None	171	108	Yes	Penicillin	No	Survived
10	Coyle 2025 [8]	18	F	Ireland	Malaise, fever, diarrhea and vomiting.	Yes	Yes	503	115	Yes	Ceftriaxone	Yes (inotrope support)	Survived
11	Reel 2025 [16]	39	F	Cuban	Fever, sore throat, wrist and chest pain	Yes	None	180	61	Yes	Azithormycin	No	Survived

CRP: C-reactive protein; ESR: erythrocyte sedimentation rate; ASOT: anti-streptolysin O titer; ITU: intensive care unit; N/A; not available.

## 5. Discussion

ARF is believed to occur about three weeks after a group A beta-hemolytic streptococcal (GAS) infection causes a type II hypersensitivity cross-reaction between streptococcal and host antigens [17]. It is a condition that exerts deleterious effects on the heart, brain, and joints. If not diagnosed early or untreated, it can graduate to rheumatic heart disease (RHD), with considerable morbidity and mortality. Its diagnosis is based on the presence of major and minor 2015 revised Jones criteria (Table 2) [18]. Our patient met the revised Jones criteria for ARF. For diagnosis, two major or one major and two minor features are needed. Our patient had arthritis and carditis as major features and two minor criteria, along with the evidence of throat infection. Incidentally, she developed ARF during the outbreak of Streptococcal-A infection in the UK.

Our patient’s case was challenging because she did not meet the criteria for ARF diagnosis at a couple of presentations to the A&E department. Arthralgia and fever falsely led clinicians to the diagnosis of a viral infection. A diagnosis of ARF was arrived at about three weeks after her first presentation, when she manifested two major criteria (carditis and polyarthritis) and two minor criteria (elevated CRP and fever). Perhaps the diagnosis could have been aided by the knowledge that there was a simultaneous Streptococcal-A infection outbreak in the UK and that her daughter recently recovered from some symptoms. The fact that her throat swab was normal was not remarkable, as only 25% of patients had a positive throat culture test at the time of diagnosis, 10+ days later [19]. It has also been shown that the ASO titer may persist for 3 months after the subsidence of the causal infection [20].

This case did not follow the typical presentation in a couple of ways. The latency of disease evolution and the initial lack of some typical clinical features made diagnosis challenging. However, such variability in typical Jones criteria has been reported in the medical literature [6]. However, and importantly, for an atypical ARF presentation, arthralgia, carditis, elevated ESR, and persistent low-grade fever are the most common symptoms [15,21]. It has been noted that arthralgia and low-grade fever may represent a milder form of ARF. Furthermore, it has been suggested that exceptions are made for the presentation of minor Jones criteria, especially when ARF is endemic in the population.

Our patient presented with carditis, which is thought to be a consequence of the overstimulation of vagal nerve endings by the rheumatic toxin [22]. This is somewhat surprising because the progression of polyarthritis or arthralgia to carditis is more common in children than adults [23]. Scientific opinions do not support the use of minor criteria like monoarthralgia and fever for the diagnosis of ARF because these symptoms are shared by other disease conditions [19].

Our SLR demonstrated that ARF remains rare in Caucasians, although incidence has been rising in recent years across regions such as Europe and North America. An observational analysis over 29 years in EU15+ countries demonstrated an increasing trend in RHD, specifically by +0.4% to + 24.7% for males and +0.6% to +11.4% for females [24]. Speculatively, this rising trend has been attributed to disparities in healthcare access for migrants, amongst other socioeconomic factors. Similar trends were observed in a 10-year review of pediatric data pertaining to acute ARF and RHD in children in the USA, with those living in more deprived communities being at more risk of severe disease [25]. Rising socioeconomic inequalities and health disparities in developed regions may account for the rising prevalence, albeit modest, in these populations. However, based on our review of cases in the literature, penicillin remains an effective mainstay of treatment, with good outcomes and rare requirements for intensive care and/or adjunctive therapies such as corticosteroids. Robust antibiotic prophylaxis for recurrent episodes has undoubtedly contributed to an overall continuing low incidence of adverse ARF outcomes in developed regions; however, lack of access to healthcare and medication for marginalized populations and the socioeconomically deprived likely in part explains the modest rise in the incidence of acute ARF and RHD in certain patient groups.

It is unusual that ARF presents for the first time in an adult, in this case at 39 years of age [15,21,26]. ARF primarily affects children from five to fifteen years of age. Presentation in adults is different, both clinically and prognostically. Presentations in children more frequently classically fit within the established Jones’ criteria. Erythema marginatum and subcutaneous nodules tend to be rare in children but remain part of the diagnostic criteria [27]. In contrast, adult presentations can be more subtle, as was the case here. Arthritis tends to be more severe and persistent, and carditis can be less common [28]. Chorea and minor criteria, such as an elevated ESR or CRP, or prolonged PR interval, may be less pronounced or more difficult to interpret, given the broader differential in adult medicine [29].

Diagnosis in adults may also be delayed, partly because clinicians may not initially consider ARF in this age group. As demonstrated by our case, echocardiography is particularly important for detecting valvular involvement or effusions that may not be clinically apparent. Delays in diagnosis, as well as pre-existing comorbidities, can lead to a poorer prognosis compared to children. Recurrences of ARF are less common in adults than in children, but when they occur, they significantly contribute to cumulative cardiac damage [30].

Epidemiological data from the USA indicate that, in children, Caucasian populations are underrepresented in hospitalizations due to ARF compared to ethnic minorities. In the Kids’ Inpatient Database (KID), Caucasian patients represented 47.8% of admissions due to ARF but 54.4% of overall pediatric hospitalizations, while Asian or Pacific Islander children were overrepresented, accounting for 6.3% of ARF admission despite comprising only 2% of pediatric admissions, a trend which has been replicated in multiple epidemiological studies [31,32]. In another study in a Hawaiian pediatric clinic, Polynesians were almost five times more likely than non-Polynesians to develop ARF [33].

Genetic studies suggest that ethnic differences in susceptibility markers may influence disease risk and presentation. Ayoub et al. [34] described in their study that genetic susceptibility to ARF differs by ethnicity, with HLA-DR2 being significantly associated with ARF in Black patients and HLA-DR4 in Caucasian patients. These associations were particularly strong for mitral insufficiency and for persistent immune response to group A streptococcal carbohydrate antigens. The findings suggested that distinct HLA class II alleles contribute to ARF risk in different ethnic groups, supporting the role of HLA-linked immune-response genes in the pathogenesis of rheumatic heart disease [34].

Studies in North Indian patients with ARF reveal that B-cell markers associated with rheumatic susceptibility differ by population. Monoclonal antibodies originally developed in North American populations exhibit high sensitivity in detecting rheumatic markers in US cohorts (up to 100%) but show significantly lower reactivity in Indian and Egyptian patients (58–70%). In contrast, a locally derived monoclonal antibody reacted with more than 10% of lymphocytes in 93% of Indian patients with chronic rheumatic fever and 87% of ARF patients, outperforming the previous antibody in the same population. These differences suggest the presence of population-specific immunological epitopes and variations in genetic background that influence susceptibility to ARF [35]. A meta-analysis of data demonstrates that specific cytokine polymorphisms, particularly TGF-β1 and IL-1β, are associated with increased RHD susceptibility. In contrast, others (e.g., TNF-α, IL-6, IL-10) show no consistent association [36]. A genome-wide genetic analysis of 1263 Aboriginal Australians demonstrated that variation in the HLA-DQA1_DQB1 region is the primary genetic risk factor for RHD in this population, with specific risk haplotypes (HLA-DQA1*0101_DQB1*0503 and HLA-DQA1*0103_DQB1*0601) being associated with increased susceptibility, while HLA-DQA1*0103_DQB1*0402 is protective [37]. Overall, larger, multiethnic genetic studies are needed to further validate these findings.

The key learning point is that a patient presenting with post-viral polyarthritis and carditis should be evaluated for ARF, irrespective of the patient’s age. Tests such as ASO-titer and throat swabs should always be considered to help diagnose the disease. Although post-viral arthritis is common, we must be vigilant about ARF during the group-A streptococcal outbreaks, as misdiagnosis could lead to long-term cardiac complications.

It is noteworthy that, previously, a minor Jones criterion was confirmation of prior streptococcal infection, but this was expunged from later revisions; this has been shown to affect diagnostic sensitivity [38]. Notwithstanding this, it might be appropriate to consider ARF with minor Jones criteria (arthralgia, low-grade fever of unknown origin, and elevated ESR/CRP/acute phase reactants), especially when there is a connection to possible contact with streptococcal infection. As outlined in our case, there was a significant delay in reaching the diagnosis of ARF; therefore, it is critical we raise awareness among clinicians that ARF can present with fever and arthralgia in adult patients in the western world, which can also mimic the symptoms of many other conditions, such as rheumatoid arthritis. The table below summarizes the clinical characteristics and risk factors of ARF and its mimickers (Table 5). 

We present a table summarizing the genetic and acquired risk factors and clinical characteristics of acute rheumatic fever (ARF) and its mimickers. Although epidemiological data are lacking in some aspects of these conditions, we summarize the key features for each condition, which should be helpful for the journal readers.

### Strengths and Limitations

This SLR is the first to focus specifically on ARF in adult Caucasians. Our SR has several limitations. The quality of evidence may be diminished due to the inclusion of case reports. Additionally, there is a possibility that some relevant studies were overlooked due to reporting bias. Nonetheless, the implementation of strict inclusion and exclusion criteria, along with a comprehensive analysis of all selected articles by several researchers, helped to reduce bias. Furthermore, due to the small number of case reports included, it was challenging to compare the characteristics of ARF.

## 6. Conclusions

ARF is rare among Caucasian adults. However, our SLR emphasizes that clinicians should consider ARF in adult Caucasian patients in the developed world who present with polyarthralgia following a sore throat. As demonstrated in our case, earlier diagnosis of ARF could prevent multiple hospital attendances.

## Figures and Tables

**Figure 3:**
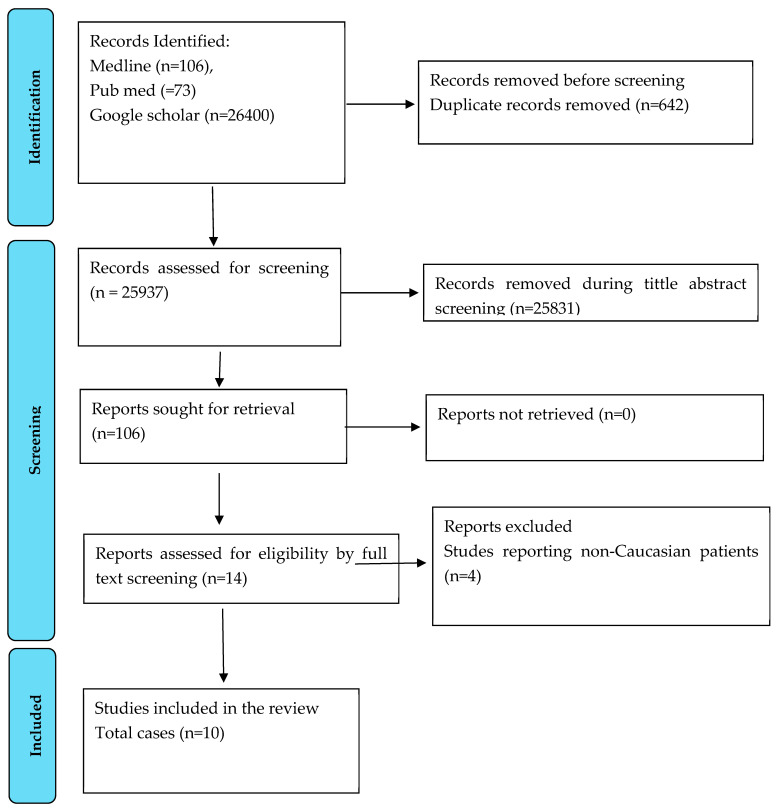
PRISMA flow diagram of data extraction of the studies included in the systematic review.

**Table 1:** Sequential investigation results at initial presentation and follow-up.

Bloods on each Hospital Admission	CRP NORMAL (<4) mg/L	Hb Normal (115–165) g/L	WCC Normal (4–11) 10*9/L	Neutrophils Normal (1.8–7.5) 10 × 9/L	Lymphocytes Normal (1–4) 10 × 9/L	Alkaline Phosphatase Norm (30–130) u/L	GGT Normal (0–39) u/L	Blood Cultures
1st visit (week 0)	171	120	6.0	4.9	0.6	142	66	Not taken
2nd visit (week 1)	191	97	4.5	3.5	0.6	210	115	Negative
3rd visit (week 2)	106	109	5.6	4.8	0.6	214	181	Negative
4th visit (week 4)	74	113	5.9	4.7	0.8	170	159	Not taken
At discharge	<4	123	3.6	2.1	1.1	78	Not taken	Not taken

CRP—C-reactive protein; Hb—hemoglobin; ALP—alkaline phosphatase; GGT—gamma-glutamyl transferase.

**Table 2:** The modified Jones diagnostic criteria of rheumatic fever.

**Major Criteria**	
**Low-risk Population**	**High-risk Population**
Carditis (clinical or subclinical) Arthritis—only polyarthritis Chorea Erythema marginatum Subcutaneous nodules	Carditis (clinical or subclinical) Arthritis—monoarthritis or polyarthritis Polyarthralgia Chorea Erythema marginatum Subcutaneous nodules
**Minor Criteria**	
**Low-risk population**	**High-risk population**
Polyarthralgia Hyperpyrexia (≥38.5 °C) ESR ≥ 60 mm/h and/or CRP ≥ 3.0 mg/dL Prolonged PR interval (after considering the differences related to age, if there is no carditis as a major criterion)	Monoarthralgia Hyperpyrexia (≥8.0 °C) ESR ≥ 30 mm/h and/or CRP ≥ 3.0 mg/dL Prolonged PR interval (after considering the differences related to age, if there is no carditis as a major criterion)

ESR—erythrocyte sedimentation rate.

**Table 3:** Echocardiogram findings pre and post-treatment.

Echocardiogram Pre-Treatment	Echocardiogram Post-Treatment after 4 Months.
A small/moderate global pericardial effusion Normal LV structure and function. Valves appeared structurally and functionally normal Mobile atrial septum, no shunt seen. No evidence of subacute bacterial endocarditis	No obvious residual pericardial effusion Normal LV structure and function No evidence of valve thickening but mild dilation of the non-coronary sinus of Valsalva and trivial AR and MR Normal RV structure and function

**Table 5:** Genetic and acquired risk factors and clinical characteristics of acute rheumatic fever (ARF) and its mimickers [21,39,40,41,42,43].

	Acute Rheumatic Fever in Children	Acute Rheumatic Fever in Adults	Adults’ Onset Still Disease	Post-Streptococcal Reactive Arthritis	Rheumatoid Arthritis
Peak age of onset (years)	5–15 yrs (rare < 3 yrs)	Rare in adults	Two peaks: 15–25 and 36–46	Bimodal peaks: 8–14 and 21–37 yrs	Typically, 40–60
Genetics	HLA-DR7, DR2/DR4; D8/17 B-cells; twin heritability ~60%	(HLA-DR7, D8/17)	HLA-DRB1	No strong HLA links; genetic link unclear	HLA-DRB1 shared epitope (DR4); twin concordance low (~12–15%)
Most common clinical features in descending order	Fever Migratory arthritis of large joints Carditis Subcutaneous nodules chorea, Erythema marginatum	Polyarthralgia (typically large joint) Fever Carditis	Quotidian fevers Salmon rash Arthritis Lymphadenopathy Hepatosplenomegaly	Non-migratory arthritis; small, axial, large joints Tenosynovitis Carditis (Rare)	Symmetric polyarthritis of small joints of hands and feet chronic erosive disease
Environmental triggers	Poor socioeconomic status.	Usually, recurrence form childhood	Triggered by viral infections, not GAS	GAS infection, but closer timing; less related to poverty	Smoking, silica, periodontal disease, obesity
Sex	Female preponderance	Female preponderance	Female predominance	Equal	Female preponderance
Most prevalent (region)	Asia, Aboriginals in Australia	N/A	Not restricted to any geographical location.	Not restricted to any geographical location.	Northern Europe and North America
Treatment	NSAIDs Penicillin Aspirin	NSAIDs Penicillin Aspirin	NSAIDs DMARDS Corticosteroids	NSAIDs Corticosteroids	NSAIDs DMARDS Corticosteroids
Complications	Rheumatic heart disease	Rheumatic heart disease	Macrophage activation syndrome	glomerulonephritis	Joint deformities Interstitial lung disease serositis

GAS—group A streptococcal infection; N/A—not applicable; NSAIDS—non-steroid anti-inflammatory drugs; DMARDS—disease-modifying antirheumatic drugs; HLA—human leucocyte antigen.

## Data Availability

The authors have the primary data and agree to allow the journal to review the data if requested. Supplementary data are available online in the *Vaccines* Journal.

## Supplementary Materials

The following supporting information can be downloaded at: https://www.mdpi.com/article/10.3390/life15071131/s1.
