# Supplementary material for: Acute Rheumatic Fever in Caucasians: A Case Report and Systematic Review
Source: Life (Basel). 2025 Jul 18;15(7):1131. doi: 10.3390/life15071131 (PMC12298489; doi:10.3390/life15071131)
Supplement: Supplementary file 1 [file life-15-01131-s001.zip › life-3606586-supplementary.pdf]

Search strategy: Acute Rheumatic Fever in Caucasians: A Case Report and Systematic Review

Medline

S1	XB rheumatic fever	**7,645**
S2	(MH "Rheumatic Fever+")	**22,459**
S3	S1 OR S2	**24,358**
S4	XB (west or western or America or north America) OR XB (west or western countries or developed countries)	**783,217**
S5	(MH "Western World+") OR (MH "Developed Countries")	**24,368**
S6	S4 OR S5	**804,089**
S7	XB (adults or adult or aged or elderly)	**2,554,338**
S8	(MH "Adult") OR (MH "Middle Aged") OR (MH "Aged, 80 and over") OR (MH "Young Adult")	**8,409,251**
S9	S7 OR S8	**9,578,541**
S10	XB (caucasian or white)	**534,958**
S11	(MH "White People")	**72,844**
S12	S10 OR S11	**564,001**
S13	S3 AND S6 AND S9 AND S12	**8**
S14	S3 AND S9 AND S12	**106**

PubMed

"rheumatic fever" AND (caucasian OR white) AND (adult OR elderly OR middle aged)	**73**

Google Scholar

"rheumatic fever" AND (caucasian OR white) AND (adult OR elderly OR middle aged) AND (case report OR case study)	**26,400**

Once all searches were done, we transferred the results into RefWorks. We then manually sifted the articles and **excluded** any that specifically dealt with *rheumatic heart disease*, those which covered *children only, those not in English and* any which did not exactly fit the criteria (eg. some results came up that included the term '*non caucasian*').

We then did some citation mining in PubMed to ensure we had not missed anything important, and the final list consisted of 14 articles for further investigation.
